# Mechanisms Accompanying Chromium Release from Concrete

**DOI:** 10.3390/ma13081891

**Published:** 2020-04-17

**Authors:** Anna Król

**Affiliations:** Department of Environmental Engineering, Faculty of Mechanical Engineering, Opole University of Technology, Prószkowska Str. 76, 45-758 Opole, Poland; a.krol@po.edu.pl

**Keywords:** chromium, leaching, concrete, Portland cement, slag cement, tank test

## Abstract

The use of mineral additives from the power and metallurgy industries in the production of building materials still raises questions about the ecological safety of such materials. These questions are particularly associated with the release of heavy metals. The article presents research related to the leaching of chromium from concretes made of Portland cement CEM I and slag cement CEM III/B (containing 75% of granulated blast furnace slag). Concrete was evaluated for leaching mechanisms that may appear during tank test over the long term (64 days). It has been presented that the dominating process associated with the leaching of chromium from both types of concrete is surface wash-off. Between the 9th and 64th day of the test, leaching of Portland cement concrete can be diffusion controlled. It has been proven that the participation of slag in the composition of concrete does not affect the level of leaching of chromium into the environment from concrete.

## 1. Introduction

In the European cement industry, waste materials are used in the production of Portland clinker and cement. At present, alternative fuels (composed of the combustible parts of industrial and municipal waste) are used on a large scale as partial substitutes for natural fuels, and waste from other industries as an alternative to raw materials naturally extracted from the environment. In addition, cement plants significantly reduce the production and use of Portland clinker (the most expensive component of cement) by introducing so-called mineral additives into the composition of cement as main components. Among the most commonly used are wastes from the power industry and metallurgy, such as silica fly ash, granulated blast furnace slag and silica dust [1,2,3]. 

They enable the adjustment of concrete properties so that this composite can meet increasingly difficult requirements in applications such as communication and underground infrastructure; engineering and mining works; and environmental protection facilities [3,4].

The use of waste in the cement industry and construction has therefore become an irreversible process, consistent with the idea of sustainable development. Over time, this solution has raised questions not only about the quality of the end products, but also about their impact on the environment. In many countries, various organizations have begun establishing a system for the environmental assessment of materials used in construction, which is to take particular account of the degree of release of heavy metals into the environment. The process of performing an environmental assessment on a material (concrete) is extremely difficult and complex. It requires consideration of factors related to the internal structure of the composite and external factors affecting concrete objects and structures in their working environment (usually in the natural environment), which may affect the release of hazardous substances. Attention should be paid to the level of release of heavy metals from concrete (concrete objects and structures), depending on the application conditions, considering the “life cycle” of the composite [5,6].

An important aspect, from the scientific point of view, is to know the processes that accompanies the release of heavy metals from building materials, especially during exposure to external factors (variable temperatures, chemically aggressive liquids, carbon dioxide). The materials may then release additional portions of heavy metals or lose their functional properties (e.g., strength properties) [7].

From a point of view of the author, it is wise to control the content of heavy metals at every “stage of life” of the mineral material, from production to application in the environment. Such a modern approach will protect the environment against the uncontrolled spread of metals. Only knowledge of the processes affecting the level of leaching can strengthen this control and predict the development of the situation under certain concrete application scenarios. A modern approach is needed to show the mechanisms that accompany release from these structures. The comprehensive leaching assessment system will give information on how concrete will behave over a long period of time in a given application environment and whether it will affect both its structure and the leaching of heavy metal. Only such a modern approach will give full control and environmental protection in the future. There are not many publications worldwide that would present this approach. Such publications most often concern only Portland cement.

The author of paper would like to emphasize the importance of research of heavy metal leaching from mineral matrices using diffusion tests; e.g., a tank test. Such tests allow the evaluation of the release in aqueous conditions of the long residence time of the sample. Construction materials are considered neutral to the environment. However, this external environment (natural or anthropogenic changed) is not without influence on concrete. During long-term exposure of concrete in water or an environment with reduced pH or under the influence of carbonation (CO_2_ impact), structural, physical and chemical changes may occur in the concrete matrix, which contributes to the release of larger portions of heavy metals. Available literature data show very few sources that would describe the relationship of leaching and the mechanisms associated with it. The author has therefore attempted to determine the mechanisms that accompany the long-term leaching from concrete. The author’s research can be used to predict long-term heavy metal leaching. Such research also would allow one to avoid the destruction of concrete during the leaching process. 

In this paper, the author presents the state of knowledge about the incorporation of chromium ions into the structures of mineral composites and the factors and processes that may affect the release of heavy metals from such matrices. The author also presented the results of her own research related to the leaching of chromium from concrete produced with Portland cement CEM I and with slag cement CEM III/B. The author found out what mechanisms accompany the leaching of chromium during the hydration process taking place at the same time and whether the composition of the matrix has a decisive effect on the chromium release level.

### 1.1. Immobilization of Chromium Ions in Cement Composites

Studies on the mechanism of heavy metal bonding and their influence on the physical and mechanical properties of mineral binders are widely discussed in the literature [8,9,10,11,12,13,14]. The authors of these works agree that each heavy metal has different characteristics in terms of the level of immobilization in the structures of mineral composites and the accompanying processes and products of hydration. Therefore, in many issues, researchers have different opinions in the literature, which gives the impression that the knowledge about the incorporation of heavy metal ions into the structures of mineral binders has not been systematized yet. 

Mattus and Gilliam [15] and de Korte and Broewers [16] placed particular emphasis on the dependence of the level of heavy metal leaching on the metals’ valencies, indicating, for example, that chromium (III) obtains a higher level of binding in the hydrated phases of mineral binding materials than chromium (VI). Very often, the environment has a reducing effect and Cr (VI) is reduced to Cr (III). The reduction of Cr (VI) to Cr (III) enables the formation of insoluble Cr (OH)_3_ hydroxide in an alkaline environment. 

It is also known that hexavalent chromium is permanently bonded by substituting the sulphate group in ettringite structures (C_3_A·3CaCrO_4_·32H_2_O). According to Glasser [17], such a reaction is also possible, but to a lesser extent, with calcium monosulfate aluminate hydrate (C_3_A·CaCrO_4_·12H_2_O). Replacement of SO_4_^2−^ by CrO_4_^2−^ is possible, but for a high degree of chromium oxidation. Therefore, immobilization of chromium is difficult and usually does not exceed 80%. Glasser [17] also claims that the relatively worse binding of hexavalent chromium may also be caused by the fact that it forms very soluble chromates.

Cr (III), on the other hand, is ingrained into the structures of phase C-S-H (hydrated calcium silicates), where substitution takes place [11,12,18]

The effect of this reaction is to stabilize the disturbed structure of the C-S-H phase and to inhibit its transition into crystalline phases [18]. The researchers do not agree on the influence of chromium ions on the hydration process. According to [19], the introduction of small amounts of chromium into the cement paste gives the effect of accelerated hydration regardless of whether Cr cation occurs at the third or sixth degree of oxidation. Wang and Vipulanandan [20] added K_2_CrO_4_ in amounts ranging from 0.5% to 5% in relation to the weight of Portland cement in binders and observed the delay of setting time of cement paste hydration along with the increase of potassium chromate additive. Tamás et al. [21] demonstrated that the introduction of trivalent chromium into cement pastes reduces the total porosity and volume of air pores, and the volume of capillary pores does not change. They claim that the reduction in pore volume may be caused by the precipitation of insoluble chromium (III) hydroxide in the space occupied by the liquid phase. However, a different thesis on the effect of chromium on the porosity of matrices is given in the paper [22]. Namely, that chromium reduces capillary porosity, but increases total porosity.

### 1.2. Processes Influencing the Leaching of Heavy Metals from Cement Composites

**Figure 1:**
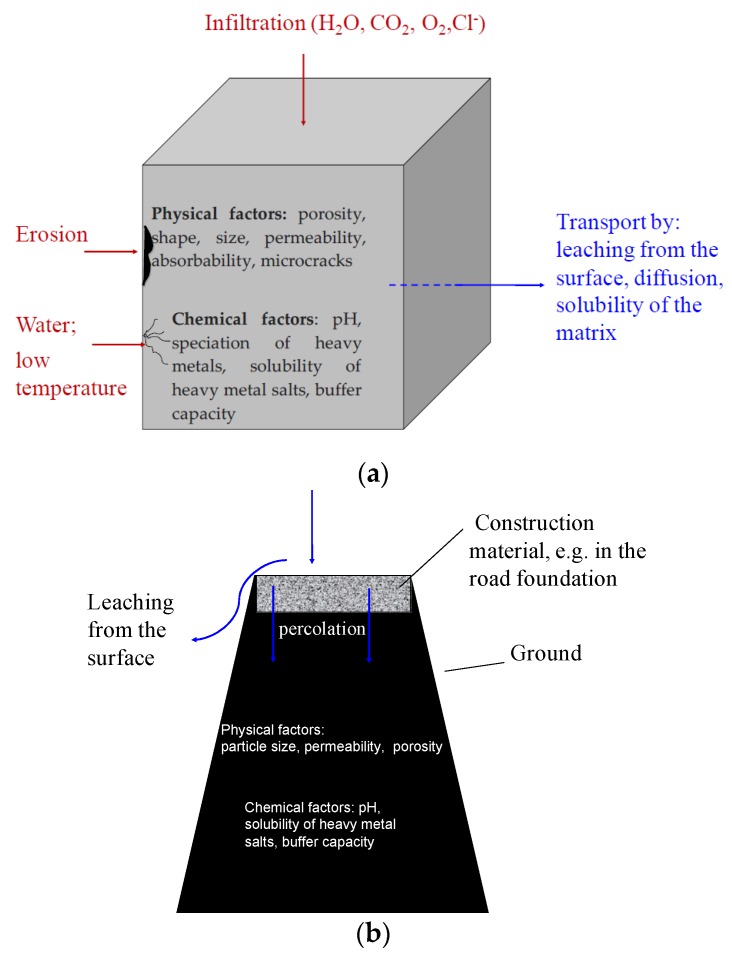
Factors and processes influencing the release of heavy metals from monolith (**a**) and crushed (**b**) mineral materials.

On the basis of the works [23,24,25], it should be concluded that the level of immobilization of heavy metals in cement matrices depends on many physical and chemical factors, which include the form of the sample (monolith or crushed sample) and environmental factors (soil, water, sewage, chemically aggressive environments, variable temperature and humidity). The level of leaching of heavy metals from a cement composite is also influenced by its composition and the water to cement ratio (w/c) a given composite was made with. The water to cement ratio (w/c); the selection of concrete components; and the amount and type of cement are the factors determining water resistance; resistance to chemical aggression; frost resistance; and strength, i.e., durability of concrete, and thus influencing the leachability of heavy metals to the environment during the whole concrete life cycle [23]. Factors and processes responsible for the release of heavy metals from construction materials are schematically presented in Figure 1. The knowledge about those factors is based on [24].

External factors include, among others, application scenario, liquid to solid ratio (L/S), time of contact with the leaching medium, ambient pH, temperature and mechanical influences (e.g., abrasion, erosion, frost).

The internal factors characterizing the tested structural material include: the porosity, thermal conductivity, shape, specific surface area, size and reactivity of the material (carbonatization susceptibility, alkalinity), and its age [12].

**Figure 2:**
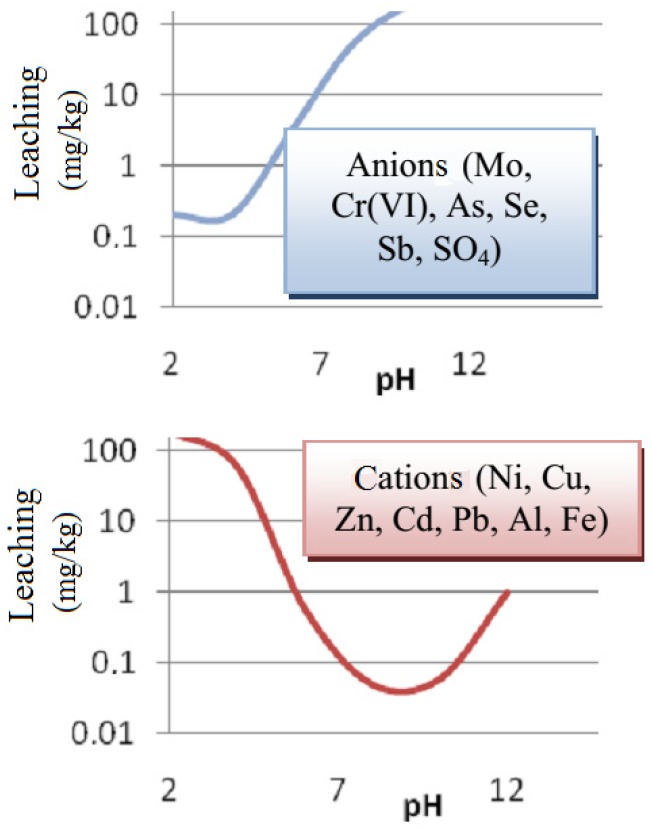
pH-dependent release of anions and cations of heavy metals.

Much attention is paid in literature to the influence of pH on the release of heavy metals [23,24,25,26]. Both the reaction of the environment surrounding the structural material (water, soil) and the reaction of the pore water of the material are important. Each heavy metal has pH-dependent solubility [27]. An example is presented in Figure 2 based on information in [27].

As a result of the decreasing pH of cement mortar (e.g., in the carbonatization process), the solubilities of heavy metals change. They usually form slightly soluble compounds in a strongly alkaline environment, whereas at a lower pH they show an increased solubility. Amphoteric metals (e.g., lead) have the lowest solubility at pH between 8 and 10 [27,28]. Studies carried out by van Gerven and others [28] have shown that elements such as magnesium, nickel and copper have the lowest solubility at pH slightly above 7. On the other hand, barium is much more easily soluble at neutral pH than at an alkaline pH. 

The results of van der Sloot’s works [29] also indicate, as a result of carbonatization (which reduces the pH of cement composites), an increased leaching of heavy metals; i.e., lead, arsenic, cobalt, zinc, molybdenum and cadmium.

## 2. Materials and Methods 

The author conducted research on the leaching of chromium from concrete on Portland cement CEM I 32,5R (abbreviation: CEM I) and on slag cement CEM III/B 32,5N-LH-HSR/NA (abbreviation: CEM III/B). The content of granulated blast furnace slag in slag cement was 75%. Table 1 shows the chemical compositions of cements, and Table 2 shows the heavy metal contents.

In the research, concrete was designed and made using both cements. The composition of the concrete mix was as follows: cement—300.0 kg/m^3^; sand—685.2 kg/m^3^; gravel 2–8 mm—600.4 kg/m^3^; gravel 8–16 mm—628.6 kg/m^3^; water—180.0 kg/m^3^; water/cement ratio (w/c)—0.6. The gravels we used were natural aggregates with a density 2.63 kg/dm^3^.

Cubes measuring 10 cm × 10 cm × 10 cm were formed from the obtained concrete mixes. After 24 h the concrete cubes were subjected to leaching tests taking into account the form of the material (integral form), time (up to 64 days of the test) and the impact of water.

**Figure 3:**
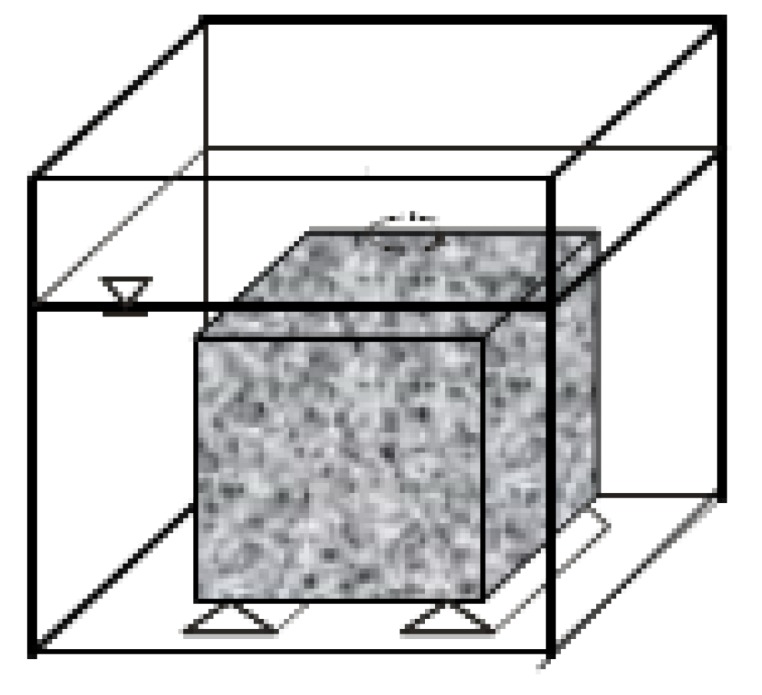
Principle of sample placement in the tank according to EA NEN 7375.

The assessment of inorganic component leaching from mineral materials in the monolithic form involves tank tests, often referred to as diffusion tests. The Netherlands Standardization Institute (NEN), European Committee for Standardization (CEN) and Environmental Protection Agency (EPA) have created a leaching test for different types of materials (building materials, stabilized waste, compacted granular materials). The most common test is based on the EA NEN 7375 standard [31]. The test procedure involves the placing of a sample with a given capacity in a vessel filled with demineralized water. The volume of water in the vessel should be two to five times greater from the volume of the sample. It is also important to place the sample at the distance of at least 2 cm from the vessel walls and to ensure that it is completely immersed in the leachant (Figure 3). The materials are then subjected to leaching over the period of 64 days. The tank test [32] allows assessing the impact of the duration of contact between the leachant and the material on the leaching of pollutants, and the analysis of the cumulative leachability of a given component per specific unit of waste surface. Thanks to the application of the tank test, it is also possible to determine the nature of the leaching; i.e., whether it is dominated by diffusion, surface wash-off, depletion or dissolution.

The test was performed at the temperature range 19–21 °C. The eluates used in the test were extracted according to the following time schedule: after 0.25, 1, 2.25, 4, 9, 16, 36 and 64 days. After each extraction of the eluate, the liquid was completely replaced and the samples were immersed in the liquid again. The eluates were filtered through the membrane filters with the pore size of 0.45 μm. The concentrations of Cr_total_ in eluates were determined with an inductively-coupled plasma-mass spectrometer (ICP MS) by Perkin Elmer. Each measurement was carried out with three repetitions holding relative standard deviation (RSD) <5%. 

## 3. Results and Discussion

In each of the eight eluates, the concentration of chromium was analyzed.

In the course of the study, the level of leaching per eluate fraction, total leachability per unit of surface area and the emerging leaching processes were determined according to [31]. 

In the course of calculations for the analyzed heavy metal, the leachability in particular fractions was determined using the Equation [31]:

(1) $$E_{i}^{*}=\frac{c_{i}\;V}{f\;A}$$

where:

$E_{i}^{*}$—leaching of the component in fraction *i* (mg/m^2^);
$c_{i}$—concentration of the component in fraction *i* (μg/dm^3^);
*V*—volume of eluate (dm^3^);
A—sample surface area (m^2^);
f—factor: 1000 (μg/mg).

Table 3 shows the leaching of chromium calculated in individual fractions. Measured cumulative leaching ($\varepsilon_{n}^{*}$) of a component was calculated (Table 3) according to the formula [31]:

(2) $$\varepsilon_{n}^{*}=\overset{n}{\underset{i=1}{\sum }}E_{i}^{*}\;\mathrm{for}\;n=1\;\mathrm{to}\;N$$

where $\varepsilon_{n}^{*}$ is the measured cumulative leaching of a component for period *n* comprising fraction *i* = 1 to *n*, in mg/m^2^ of sample surface area; $E_{i}^{*}$ is the measured leaching of the component in fraction *i*, in mg/m^2^; and *N* is the total number of leachant replenishment periods.

Derived cumulative leaching $\varepsilon_{n}$ of a component was calculated using the formula [31]:

(3) $$\varepsilon_{n}=E_{i}^{*}\frac{\sqrt{t_{i}}}{\sqrt{t_{i}}-\sqrt{t_{i-1}}}$$

where $\varepsilon_{n}$ is the derived cumulative leaching of a component for period *n* comprising fraction *i* = 1 to *n*, in mg/m^2^ of sample surface area; $E_{i}^{*}$ is the measured leaching of the component in fraction *i*, in mg/m^2^; *t_i_* is the replenishment time of fraction *i*, in *s*; and *t*_*i*−1_ is the replenishment time of fraction *i*−1, in *s*.

Based on the above calculations, it can be determined which mechanisms accompany heavy metal leaching; i.e., whether each is dominated by diffusion (DIF) or other mechanisms, such as:

—Surface wash-off (SWO);
—Depletion (DEP);
—Dissolution (DIS);
—Delayed diffusion or dissolution (DDD).

**Figure 4:**
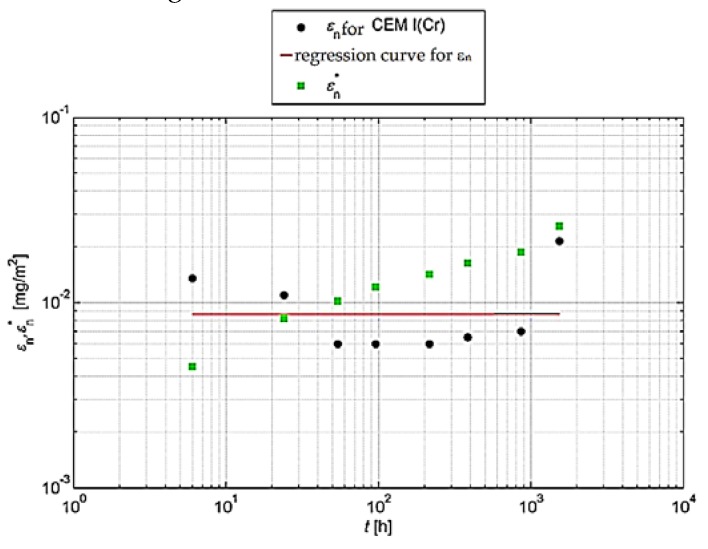
Cumulative leaching $\varepsilon_{n}$ of chromium with a determined regression curve for concrete with CEM I.

**Figure 5:**
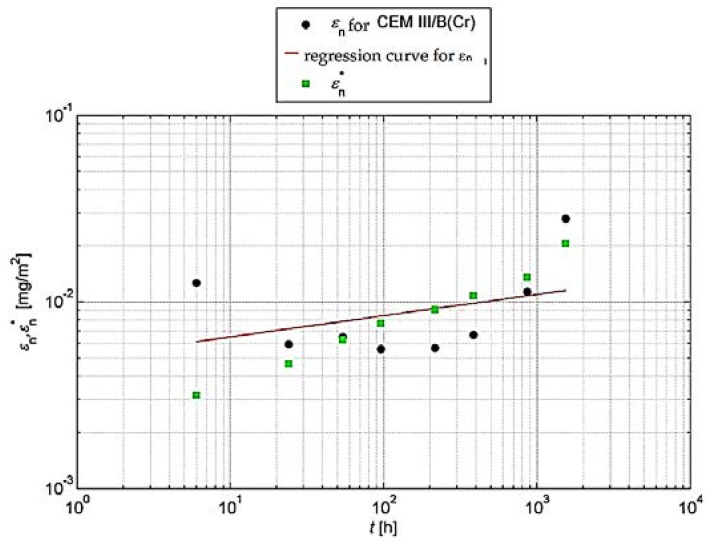
Cumulative leaching $\varepsilon_{n}$ of chromium with a determined regression curve for concrete with CEM III/B.

For such an analysis, it is recommended [31] that the cumulative leaching ($\varepsilon_{n}^{*}$ and $\varepsilon_{n}$) should be shown graphically. For this purpose, the logarithm of the cumulative leaching $\varepsilon_{n}$ obtained in relation to the time logarithm *t_i_* for n = 1 to N should be plotted in order to visually assess the measurements obtained. In the same graph, the logarithm of the calculated total leaching $\varepsilon_{n}^{*}$ should be plotted. Graphical analyses are shown in Figure 4 and Figure 5.

The eluate fractions obtained and tested during periods 1–8 should be divided into increments long enough to recognize the mechanisms involved in release of heavy metals. Eluate fractions collected in periods 1–8 should be grouped into the ranges like shown in Table 4 [31].

The *CF_a−b_* concentration factor (Equation (4)), slope (rc) of linear regression line of log ε versus log *t* and the standard deviation (sd_rc_) shall be determined for a given heavy metal under assessment and for each of the ranges identified.

(4) $$CF_{a-b}=\frac{\mathrm{Mean}\;\mathrm{concentration}\;\mathrm{in}\;\mathrm{the}\;\mathrm{increment}}{\mathrm{Lowest}\;\mathrm{limit}\;\mathrm{of}\;\mathrm{determination}\;\mathrm{of}\;a\;\mathrm{heavy}\;\mathrm{metal}}$$

On the basis of the slope of the regression function in individual increments (Table 5), it can be specified which mechanisms control the release of heavy metals from the test sample. 

**Figure 6:**
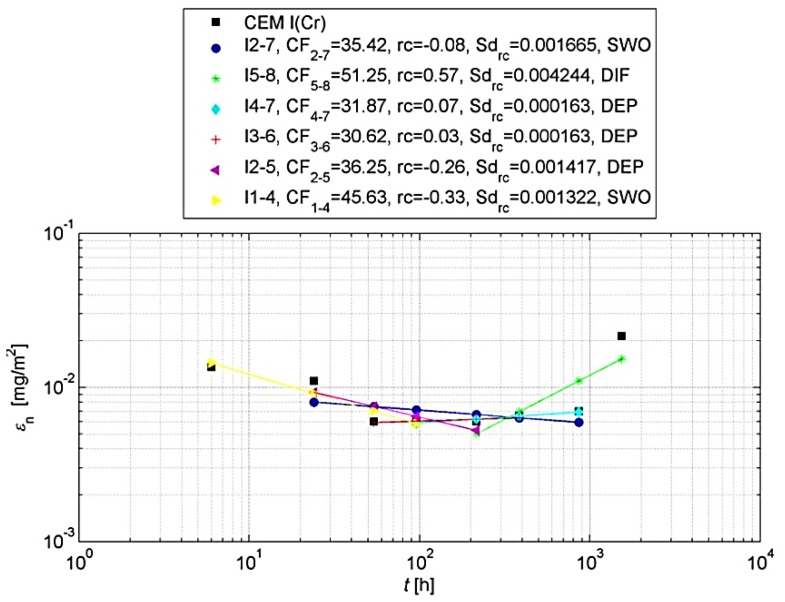
Cumulative leaching $\varepsilon_{n}$ of chromium with determined regression curve in increments, for concrete with CEM I.

**Figure 7:**
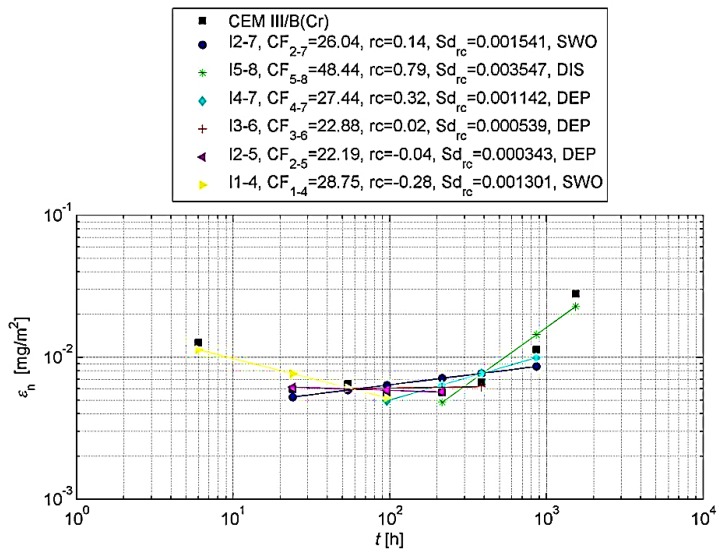
Cumulative leaching $\varepsilon_{n}$ of chromium with determined regression curve in increments, for concrete with CEM III/B.

Graphical representations of the results obtained for chromium are shown in Figure 6 and Figure 7.

On the basis of the above analyses, it can be concluded that the leachability of chromium from concrete with Portland cement and with slag cement is not controlled during the entire test cycle by one leaching mechanism. However, it can be claimed that the release of chromium in both the longest (increments 2–7) and the shortest and earliest test period (increment 1–4) occurs by surface wash-off (SWO). The tests also showed that the matrix with CEM I does not dissolve, which means that the matrix did not deteriorate during long (64 days) chromium leaching tests. In later test periods (between the 9th and 64th day of the test) the leaching of chromium from concrete may be accompanied by diffusion processes.

Dissolution occurs in the case of concrete with CEM III/B (increment 5−8), which may indicate the destruction of the concrete during long-term exposure to water. However, during this time there are no increased doses of released chromium per area unit, compared to concrete with Portland cement (Table 3). Studies have shown that the achieved level of chromium release from concrete with CEM III/B can be even lower compared to concrete on Portland cement. This may be related to the caulking structure of concrete affected by granulated blast furnace slag. Composites made of slag cement are characterized by a smaller number of capillary pores, and greater tightness of the structure, and thus a reduction in the permeability and penetration of aggressive substances and water [32,33].

## 4. Conclusions

The use of alternative fuels and by-products from the power and metallurgy industries as valuable components of clinkers, cements and mineral composites has become in the last twenty years a fully intentional activity of the cement and construction industry, consistent with the idea of sustainable development. This has led to the development of a wide range of industrial by-products that are able to meet the challenges of modern construction. On the other hand, however, the system of environmental assessments has become extremely topical, which should cover the difficult subject of releasing heavy metals from mineral composites into the environment through research procedures. Concrete is commonly present in the environments surrounding people. Therefore, it must not affect the quality of the environment with which it is in contact, and particularly, it must not impair human health.

The paper presents considerations on the mechanisms accompanying the leaching of heavy metals from building materials, focusing on the release of chromium from concrete produced with Portland cement and slag cement. 

The results of chromium leaching from concrete seasoned up to 64 days were obtained. Processes accompanying the release of chromium from concrete matrices were evaluated. It was found that the dominant process which controls the leaching of chromium from the analyzed concretes is surface wash-off. It has also been proven that the use of large amounts of granulated blast furnace slag in the cement composition does not release additional portions of chromium from concretes in long contact with water. Concrete with slag cement can be influenced by dissolution. In practice, therefore, more tight concrete of this type should be used (e.g., by lowering the w/c ratio of the concrete), which would allow obtaining a compact structure with a lower capillary content. This can protect slag concrete from dissolving during long term contact with water. As a result, this will contribute to not releasing further portions of chromium into the environment.

There is not much research in the literature on the mechanisms controlling the leaching process; hence, the author took up this subject. However, this issue should be further developed considering other concrete exposure conditions and other kinds of concrete. If such complex tests are carried out, it will be possible to plot the matrices of the use of individual concretes in various application scenarios, so additional heavy metal concentrations are not released.

## Figures and Tables

**Table 1:** Chemical compositions of cements used in the tests.

Component	Content [Weight %]	
	CEM I	CEM III/B
Ignition losses	3.46	0.42
Insoluble parts	0.44	0.48
CaO	64.60	49.75
SiO_2_	19.20	32.92
Al_2_O_3_	4.69	6.96
Fe_2_O_3_	3.04	1.80
MgO	1.22	5.04
SO_3_	2.65	1.44
K_2_O	0.81	0.83
Na_2_O	0.09	0.28
Cl^−^	0.047	0.067

**Table 2:** Heavy metal contents of cements used in the tests [30].

Heavy Metal	Content [mg/kg]	
	CEM I	CEM III B
Cr	54	31
Zn	316	105
Cd	<1	<1
Pb	24	39
Co	7	3
Ni	18	11
Mn	288	1638
V	34	31
Cu	60	27
As	6	0,3
Hg	<0.08	0.08
Tl	<5	<5

**Table 3:** Chromium leaching out of concrete with CEM I and CEM III/B cement.

Eluate Fraction	Time [h]	Chromium Leachability $E_{i}^{*}$ [mg/m^2^]		Measured Cumulative Leachability of Chromium $\varepsilon_{n}^{*}$ [mg/m^2^]	
		CEM I Concrete	CEM III/B Concrete	CEM I Concrete	CEM III/B Concrete
1	6	0.0045	0.0031	0.0045	0.0032
2	24	0.0037	0.0015	0.0082	0.0046
3	54	0.0020	0.0016	0.0102	0.0063
4	96	0.0020	0.0014	0.0122	0.0077
5	216	0.0020	0.0014	0.0142	0.0091
6	384	0.0022	0.0017	0.0163	0.0107
7	846	0.0023	0.0028	0.0187	0.0136
8	1536	0.0072	0.0070	0.0258	0.0206

**Table 4:** Ranges of eluates and increments.

Number of Range	Eluate Fraction	Increments a−b
1	Fractions 2 to 7	Increment 2−7
2	Fractions 5 to 8	Increment 5−8
3	Fractions 4 to 7	Increment 4−7
4	Fractions 3 to 6	Increment 3−6
5	Fractions 2 to 5	Increment 2−5
6	Fractions 1 to 5	Increment 1−4

**Table 5:** Interpretation of rc slopes within individual increments [31].

Increment a−b	Slope, rc		
	≤0.35	> 0.35 i ≤ 0.65	>0.65
Increment 2−7	Surface Wash-off (SWO)	Diffusion (DIF)	Dissolution (DIS)
Increment 5−8	Depletion (DEP)	Diffusion (DIF)	Dissolution (DIS)
Increment 4−7	Depletion (DEP)	Diffusion (DIF)	Dissolution (DIS)
Increment 3−6	Depletion (DEP)	Diffusion (DIF)	Dissolution (DIS)
Increment 2−5	Depletion (DEP)	Diffusion (DIF)	Dissolution (DIS)
Increment 1−4	Surface Wash-off (SWO)	Diffusion (DIF)	Delayed diffusion or dissolution (DDD)
